# Comparative transcriptome analysis of whiteflies raised on *cotton leaf curl Multan virus*-infected cotton plants

**DOI:** 10.3389/fvets.2024.1417590

**Published:** 2024-08-28

**Authors:** Ting Chen, Yanbo Jia, Jie Chen, Guojun Qi

**Affiliations:** Guangdong Provincial Key Laboratory of High Technology for Plant Protection /Plant Protection Research Institute, Guangdong Academy of Agricultural Sciences, Guangzhou, China

**Keywords:** *cotton leaf curl Multan virus*, *Bemisia tabaci*, cryptic species, transcriptome, expression profiles

## Abstract

*Cotton leaf curl Multan virus* (CLCuMuV), a serious viral disease causative agent in cotton plants in South Asia, is transmitted by the *Bemisia tabaci* cryptic species complex in a persistent circulative manner. A previous study indicated that Asia II-7 whiteflies could transmit CLCuMuV, while Mediterranean (MED) whiteflies failed to transmit CLCuMuV. However, little is known about the genes involved in this process. In this study, Asia II-7 and MED *B. tabaci* were utilized to determine transcriptomic responses after 48 h of acquisition access periods (AAPs). Result of Illumina sequencing revealed that, 14,213 and 8,986 differentially expressed genes (DEGs) were identified. Furthermore, DEGs related to the immune system and metabolism of Asia II-7 and MED in response to CLCuMuV-infected plants were identified and analyzed using Gene Ontologies (GO) and the Kyoto Encyclopedia of Genes and Genomes (KEGG), and the number of related DEGs in MED was lower than that of Asia II-7. The most abundant groups of DEGs between both viruliferous and aviruliferous whitefly species were the zf-C2H2 family of transcription factors (TFs). Notably, in comparison to viruliferous MED, Asia II-7 exhibited more DEGs related to cathepsin biosynthesis. Overall, this study provides the basic information for investigating the molecular mechanism of how begomoviruses affect *B. tabaci* metabolism and immune response either as vector cryptic species or non-vector species.

## 1 Introduction

Host plant-mediated interaction between viruses and insects plays a vital role in the epidemiology of plant diseases because plant diseases caused by begomoviruses limit the production of many economic crops, such as tomato (1), cassava (2), and cotton (3), causing tremendous losses of agricultural crops worldwide (4, 5). Begomoviruses are transmitted in a persistent circulative manner by whiteflies *Bemisia tabaci*, where midgut and primary salivary glands have been identified as barriers in the circulation in the vector body (6–8). Overall, the interaction between plants, begomoviruses, and whiteflies is associated with various molecular mechanisms (9, 10).

*Cotton leaf curl Multan virus* (CLCuMuV) is a serious and economically important viral disease agent in cotton and ornamental plants in many regions of the world (11). In China, CLCuMuV was first detected in Guangzhou (12) and had rapidly spread to many provinces including Guangxi, Hainan, Fujian, and Jiangsu (13, 14). Among 39 known cryptic species of *B. tabaci*, two indigenous species, Asia II-1 and Asia II-7, are possibly the most efficient vectors of CLCuMuV (15, 16). On the contrary, two invasive species, Middle East Asia Minor 1 (MEAM1) and Mediterranean (MED), have been proven to be unable to transmit CLCuMuV. The previous study also revealed that after being ingested by whiteflies, CLCuMuV had a poorer capacity to cross the midguts of MEAM1 and MED than AsiaII1 (16). Additionally, when begomoviruses transmit from the midgut lumen to the hemolymph, they regulate numerous genes in whiteflies (17).

Previous reports compared the gene expression of whiteflies fed on healthy plants and those fed on virus-infected plants to unravel the mechanism of whiteflies and virus interaction (18–20). The expression of metabolism- and immune reaction-related genes of whiteflies was altered after the acquisition of *Tomato yellow leaf curl China virus* (TYLCCNV) and *Tomato yellow leaf curl virus* (TYLCV) (18, 21). However, to the best of our knowledge, few studies focused on the differences in gene expression between whitefly vector species and non-vector species, which could help reveal the interaction between begomovirus and whitefly.

In this study, we collected aviruliferous and viruliferous Asia II-7 and MED of *B. tabaci,* respectively, and compared all the genes to elucidate the interaction underlying different combinations of whiteflies and begomoviruses. Comparative results of aviruliferous/viruliferous Asia II-7 and aviruliferous/viruliferous MED were obtained. Overall, this study provides the theoretical foundation to explore the molecular mechanism of how begomoviruses affect the molecular response of whiteflies either as vectors or non-vectors and select candidate genes involved in the immune response and metabolism of whiteflies for future functional studies.

## 2 Materials and methods

### 2.1 Insects, plants, and viruses

Species of whiteflies, Asia II-7 and MED colony (*mtCOI* GeneBank accession code: KM821541 and GQ371165), and two cotton cultivars, namely, *Gossypium hirsutum* L. cv. Zhongmian40 and cv. 112–2, were used for experiments. Clones of CLCuMuV (accession number KP762786) and CLCuMuB (KP762787) were obtained from the plant protection institute, Guangdong Academy of Agricultural Sciences.

### 2.2 Preparation of whitefly samples

Cultures of whitefly Asia II-7 and MED were reared on uninfected cotton plants (*G. hirsutum* cv. Zhongmian40) in insect-proof cages separately. For experiments, uninfected cotton plants of Zhongmian40 were cultivated to 7–8 true leaf stages when used. For the preparation of CLCuMuV-infected plants, cotton plants of 112–2 were first cultivated to 2–3 true leaf stage when virus inoculation was conducted, and then, the virus-inoculated plants were further cultivated to 7–8 true leaf stage when used. The status of virus infection of these plants with typical symptoms was verified by PCR analysis, and the primers used for testing are presented in Supplementary Table S1. To obtain whiteflies fed on uninfected and CLCuMuV-infected plants, whitefly adults from Asia II-7 and MED cultures were collected 7–8 d post-emergence and then placed on uninfected and CLCuMuV-infected cotton plants of 112–2, respectively. The whiteflies were then collected after 48 h of AAP. For each of the four treatments, e.g., aviruliferous Asia II-7 (AA), viruliferous Asia II-7 (IU), aviruliferous MED (AM), and viruliferous MED (VM), three biological replicates in three separate cages were conducted. All whitefly cultures of the four treatments were reared in cages at 26 ± 1°C and 16 h light/8 h darkness.

### 2.3 Transcriptome sequencing and analysis

For each replicate sampling, 200 individual whiteflies were collected into 1.5 mL microcentrifuge tubes and then flash frozen using liquid nitrogen and stored at −80°C.

The total RNA of each sample was extracted using Trizol reagent (purchased from Takara, Japan), following the manufacturer’s instructions. Overall, 1.5 μg of RNA per sample was used as input material for RNA sample preparation. As described by Li et al. (17), RNA-Seq libraries were constructed using RNA extracted from whiteflies and sequenced by HiSeq 2,500 (Illumina, Inc. USA), and RNA-Seq data were analyzed as described by Ding et al. (22). RNA-Seq raw reads were processed and normalized to fragments per kilobase million mapped reads (FPKM), and differential expression analysis was performed using edger. The resulting *p*-values were adjusted for multiple testing using a false discovery rate (FDR). For the identification of differentially expressed genes (DEGs), the following cutoff parameters were used: the threshold of Q-value (adjusted *p*-value) of ≤0.001 and log_2_ (fold change) ≥1. The deduced protein sequences of DEGs were used to generate Gene Ontology (GO) and Kyoto Encyclopedia of Genes and Genomes (KEGG) functional classifications. Data were analyzed by Novogene (Tianjin, China).

### 2.4 Transcriptome sequencing and analysis

Seven DEGs were selected for qPCR analysis to validate the data of RNA-Seq. Total RNA was extracted from Asia II-7 and MED whiteflies fed on either CLCuMuV-infected or uninfected cotton plants for 48 h using the same methods and kits of RNA-Seq. In total, 0.5 μg of total RNA was reversely transcribed using the HiScript 1st Strand cDNA Synthesis Kit (QIAGEN, USA). The qPCR was performed on the CFX96 TM Real-Time PCR Detection System (Bio-Rad, USA) with SYBR Premix ExTaq II (Takara, Japan). β-actin was used as the reference gene (20). The relative expression levels of DEGs were calculated by the 2^-ΔΔCt^ method (23). The qPCR primers of targeted and reference genes are presented in Table 1.

**Table 1:** CLCuMuV-regulated candidate DEGs related to the CTS family in Asia II-7 and MED whiteflies.

Gene family	Gene ID	VA vs. AA		VM vs. AM	
		Up or down	log_2_FC	UP OR DOWN	log_2_FC
CTSB	Cluster-35353.35090	Up	4.13	Up	1.36
	Cluster-35353.61151	Up	3.99	Up	2.06
	Cluster-35353.34404	Up	7.48	Up	2.65
	Cluster-35353.35601	Up	2.54	False	0.02
	Cluster-35353.42127	Up	4.3	False	1.61
	Cluster-35353.66030	Down	−1.73	Down	−1.73
	Cluster-35353.65744	Up	2.35	False	1.29
	Cluster-35353.28899	Up	3.08	Up	1.32
	Cluster-35353.62637	Up	4.23	Up	5.82
	Cluster-35353.19909	Up	5.53	False	0.91
	Cluster-35353.45670	Up	3.75	False	0.84
	Cluster-35353.60473	Up	1.94	Up	3.11
	Cluster-35353.55424	Up	1.24	Up	1.86
	Cluster-35353.56882	Up	2.57	False	0.24
	Cluster-35353.20118	Up	4.45	Up	2.51
	Cluster-35353.31497	Up	2.05	False	0.28
	Cluster-35353.41624	Up	3.83	Up	3.31
	Cluster-35353.41620	Up	1.21	Up	1.87
	Cluster-35353.54838	Up	3.37	False	2.46
	Cluster-35353.50921	Up	2.92	False	−0.23
	Cluster-35353.25540	Up	2.15	False	−0.36
	Cluster-35353.44494	Up	3.03	False	1.19
	Cluster-35353.42129	Up	4	False	1.09
	Cluster-35353.35086	Up	2.36	False	0.01
	Cluster-35353.63511	Up	3.41	Up	3.8
	Cluster-35353.50232	Up	5.71	Up	2.27
	Cluster-35353.43361	Up	2.21	False	0.35
CTSF	Cluster-35353.25183	Up	3.21	False	0.87
	Cluster-35353.64940	Up	6.55	Up	3.3
	Cluster-35353.50214	Up	2.05	False	0.55
	Cluster-35353.24966	Up	1.16	False	1.44
	Cluster-35353.25579	Up	2.84	False	0.73
	Cluster-35353.36510	Up	1.27	Up	1.2
	Cluster-35353.36497	Up	2.98	False	0.78
	Cluster-35353.35975	Up	1.57	False	0.39
	Cluster-35353.29929	Up	2.67	False	0.17
CTSH	Cluster-35353.45026	Up	8.74	Up	2.86
CTSK	Cluster-35353.37946	Up	7.95	Up	4.49
	Cluster-32364.1	Down	−2.13	False	−0.8
	Cluster-35353.23691	Up	1.72	False	0.51
CTSL	Cluster-35353.23628	Up	3.18	Up	3.05
	Cluster-35353.64941	Up	5.44	False	2.14
	Cluster-35353.22822	Up	1.51	Up	1.93
	Cluster-35353.42930	Up	3.4	False	−0.08
CTSW	Cluster-35353.65784	Down	−1.92	Down	−4.1

AA, aviruliferous Asia II-7; VA, viruliferousAsia II-7; AM, aviruliferous MED; VM, viruliferous MED.

### 2.5 CLCuMuV detection by polymerase chain reaction (PCR)

The DNA of the 10 whitefly adults of viruliferous and aviruliferous groups of Asia II-7 and MEAM1 was extracted with an Easy Pure Genomic DNA Kit (Trans Gen Biotech, Beijing, China), respectively. The DNA samples were stored at −20°C. The primer sequences used for CLCuMuV detection were forward (5’-CAGGAAGCAGGAAAATACGAGA-3′)/reverse (5’-TGGCAGTCCAACACAAAATACG-3′), with an amplicon of 831 bp. PCR was performed using 25 μL of a PCR mixture containing 2 μL of template DNA, 12.5 μL of 2xTaq Plus Master Mix (Vazyme, Nanjing, China), 1 μL of a 10 μM solution of each primer, and 8.5 μL of sterile double-distilled water. PCR amplification was performed with a GeneAmp 9,600 PCR system (Perkin–Elmer) using the following temperature program: 1 cycle of denaturation for 4 min at 94°C; 35 cycles of melting at 94°C for 45 s, annealing at 52°C for 45 s, and elongation at 72°C for 1 min; and a final extension round at 72°C for 10 min. The PCR amplicons were separated electrophoretically onto a 1% agarose gel (pulsed-field-certified agarose; Bio-Rad Laboratories AG).

## 3 Results

### 3.1 Transcriptome overview

**Figure 1:**
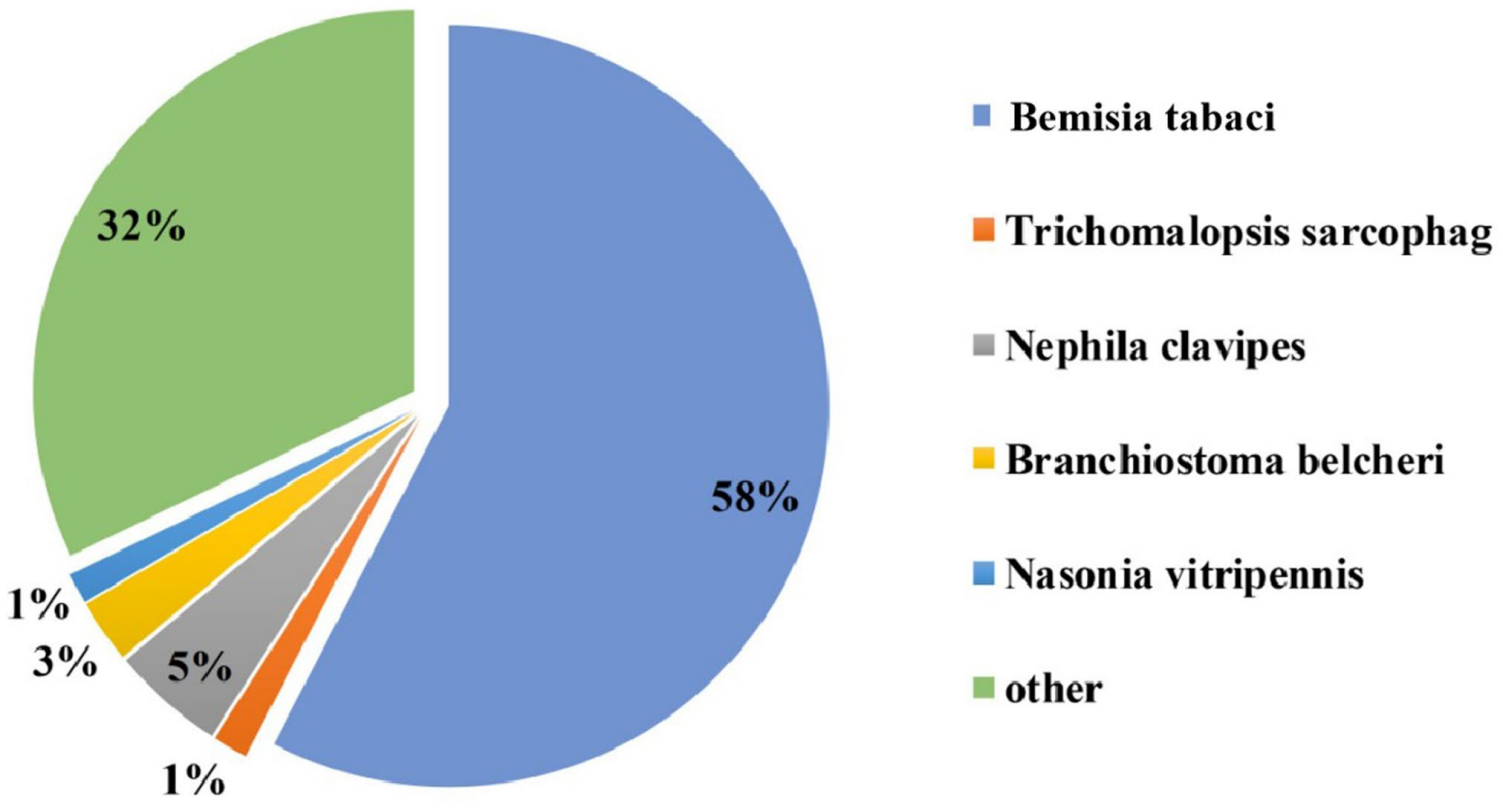
Nr homologous species distribution. All the genes were searched in the Nr nucleotide database using BLASTX.

The acquisition of CLCuMuV by Asia II-7 and MED was first confirmed by PCR (Fig. S1). Then, the transcriptome of cryptic species Asia II-7 and MED of *B. tabaci* fed on CLCuMuV-infected and healthy cotton plants was sequenced and compared. A total of 74.28 Gb of clean data and 117,542 unigenes were identified with annotation information, with a mean length of 952 bp of a gene. The N50 was 1,392 bp, and the Q30 (the probability of an incorrect base call 1 in 1,000 times) was more than 92.09% (Supplementary Table S2). A total of 39,977 genes showed a significant hit stnr_INV, 57.6% of which had a top hit to *Bemisia tabaci* followed by *Nephila clavipes* (4.8%) (Figure 1).

### 3.2 DEGs in cryptic species Asia II-7 and MED of *B. tabaci* exposed to CLCuMuV

**Figure 2:**
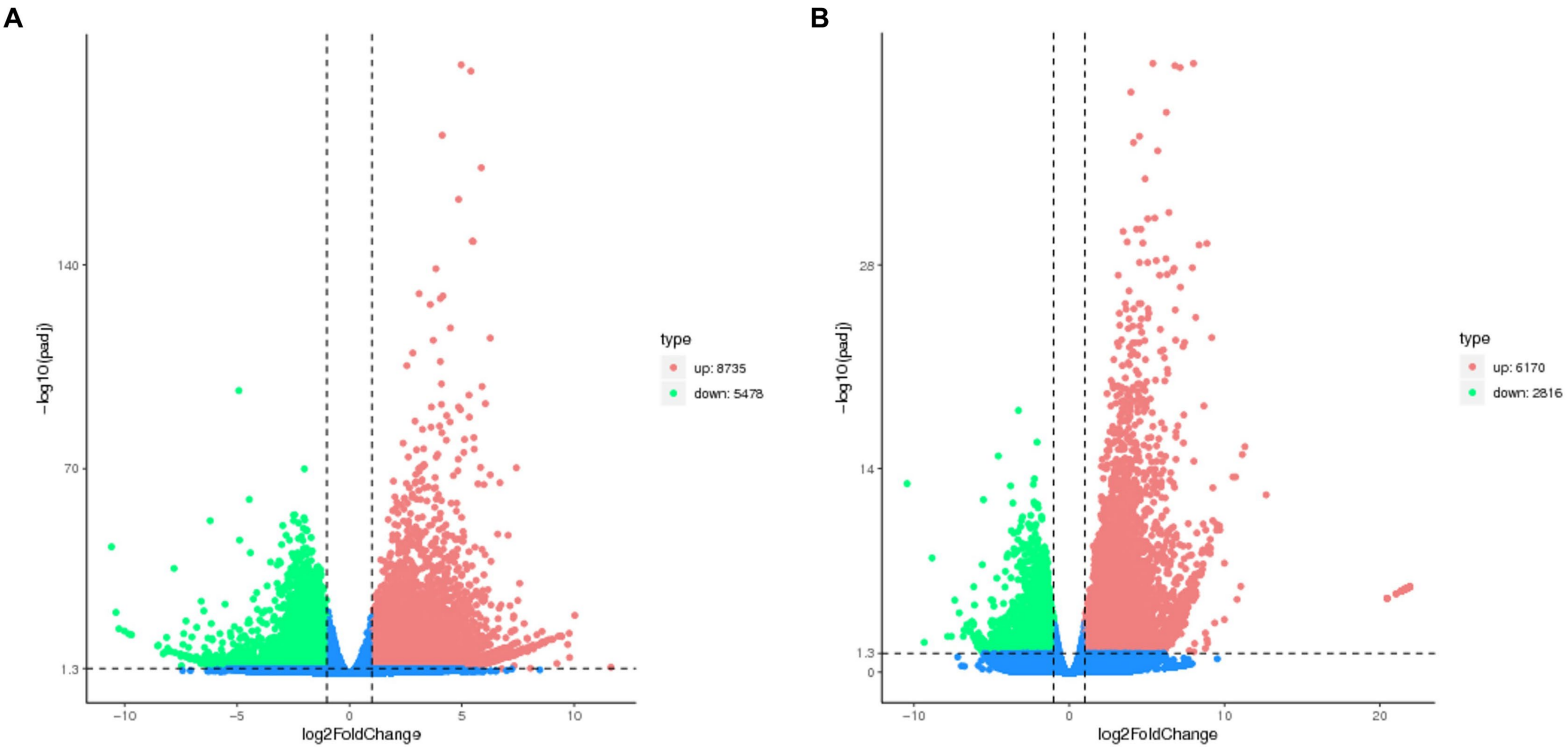
Volcano plot of DEGs of AA vs. VA **(A)** and AM vs. VM **(B)**. Non-DEGs are shown as blue dots. Upregulated and downregulated DEGs are shown as red and green dots, respectively. AA, aviruliferous Asia II-7; VA, viruliferous Asia II-7; AM, aviruliferous MED; and VM, viruliferous MED.

**Figure 3:**
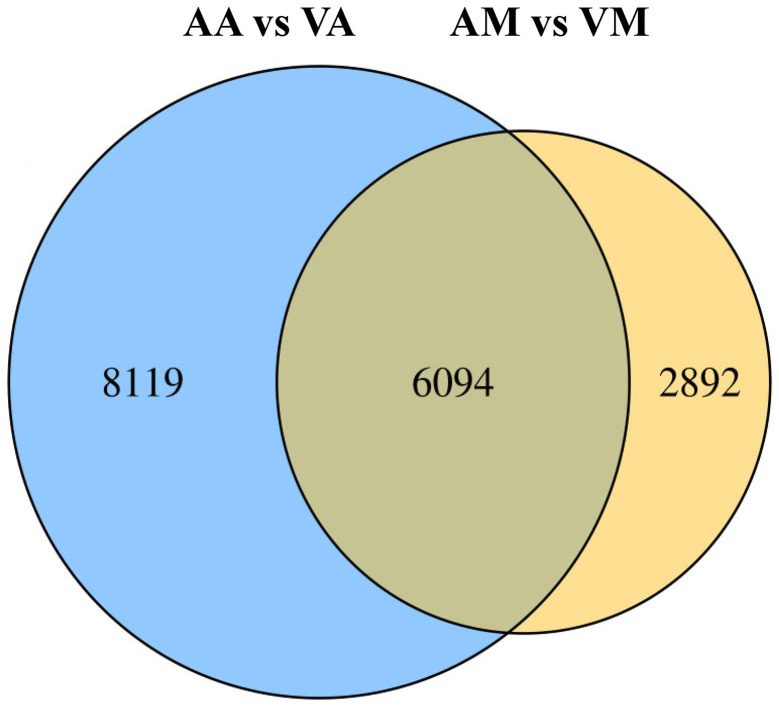
Venn diagram of DEGs of viruliferous/aviruliferous whiteflies. AA, aviruliferous Asia II-7; VA, viruliferous Asia II-7; AM, aviruliferous MED; and VM, viruliferous MED.

The number of DEGs in cryptic species Asia II-7 and MED of *B. tabaci* exposed to CLCuMuV infection was 14,213 and 8,986, respectively. The changed ratios of most DEGs in the two groups were between 2^−5^ and 2^5^ (Figure 2). Among all the 14,213 DEGs in viruliferous Asia II-7 and aviruliferous whiteflies, 8,735 were upregulated, while 5,478 were downregulated. As for the DEGs in viruliferous MED and aviruliferous whiteflies, 6,170 were upregulated, while 2,816 were downregulated. Moreover, aviruliferous/viruliferous Asia II-7 and aviruliferous/viruliferous MED shared 6,094 DEGs, with specific DEGs of 8,119 and 2,892, respectively (Figure 3).

**Figure 4:**
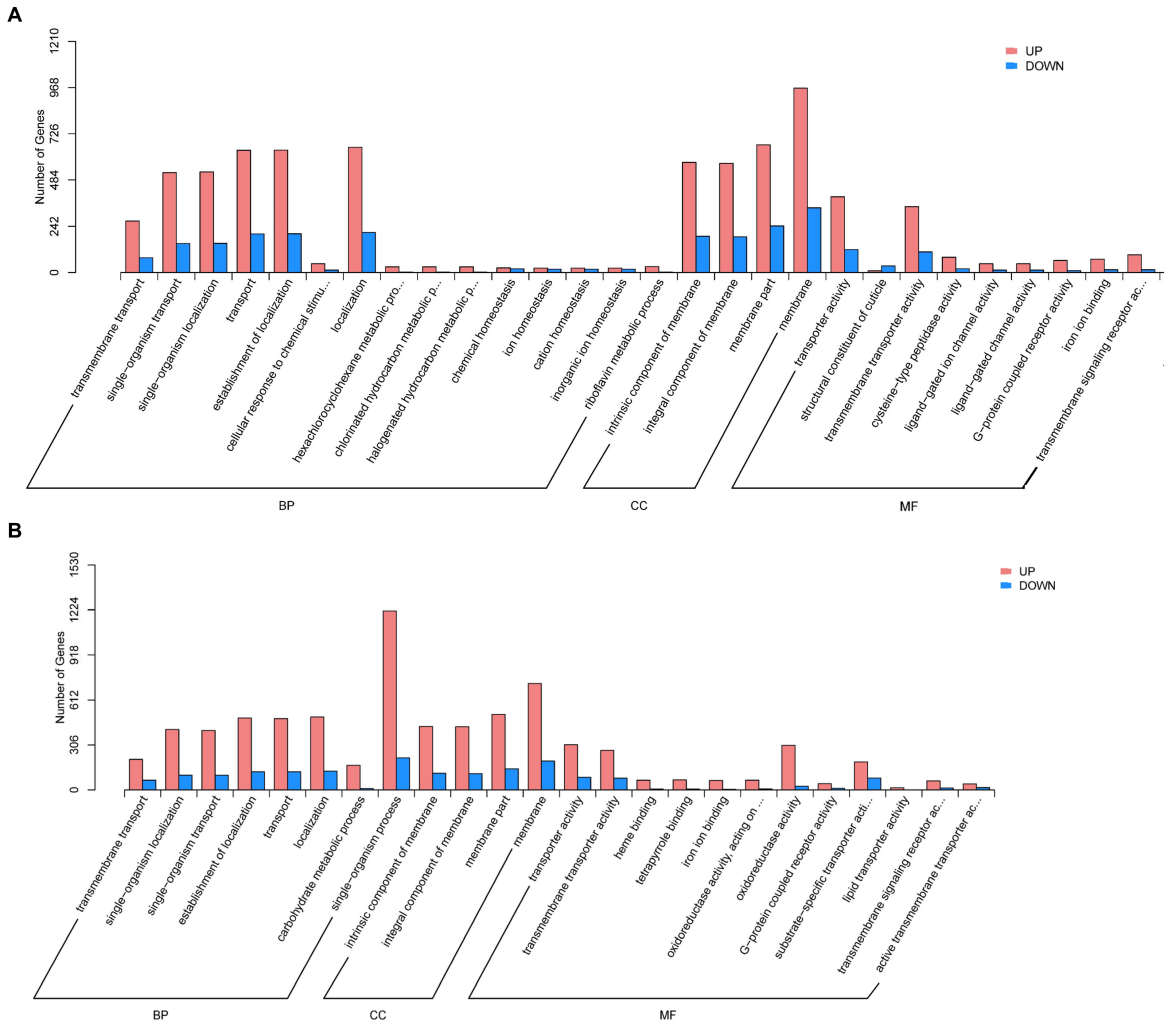
Gene ontology analysis of AA vs. VA **(A)** and AM vs. VM **(B)**. The bar chart shows the distribution of the corresponding GO terms (*p* < 0.05). Different colors represent different GO categories. AA, aviruliferous Asia II-7; VA, viruliferous Asia II-7; AM, aviruliferous MED; and VM, viruliferous MED.

The GO analysis revealed that DEGs of aviruliferous/viruliferous Asia II-7 were associated with 34 GO items in three categories, i.e., biological process, molecular function, and cellular component (Figure 4). In the biological process category, dominant items were localization (865), the establishment of localization (843), transport (841), cellular process (864), and single-organism localization (679). In the molecular function category, transporter activity (517) and transmembrane transporter activity (453) were the dominant items. Moreover, in the cellular component category, the annotated genes were mostly involved in the membrane (1304) and membrane part (912). Finally, DEGs of aviruliferous/viruliferous MED were associated with 24 GO items, where dominant items were single-organism process (1434), membrane (922), membrane part (657), localization (624), the establishment of localization (612), and transport (608) (Figure 4).

**Figure 5:**
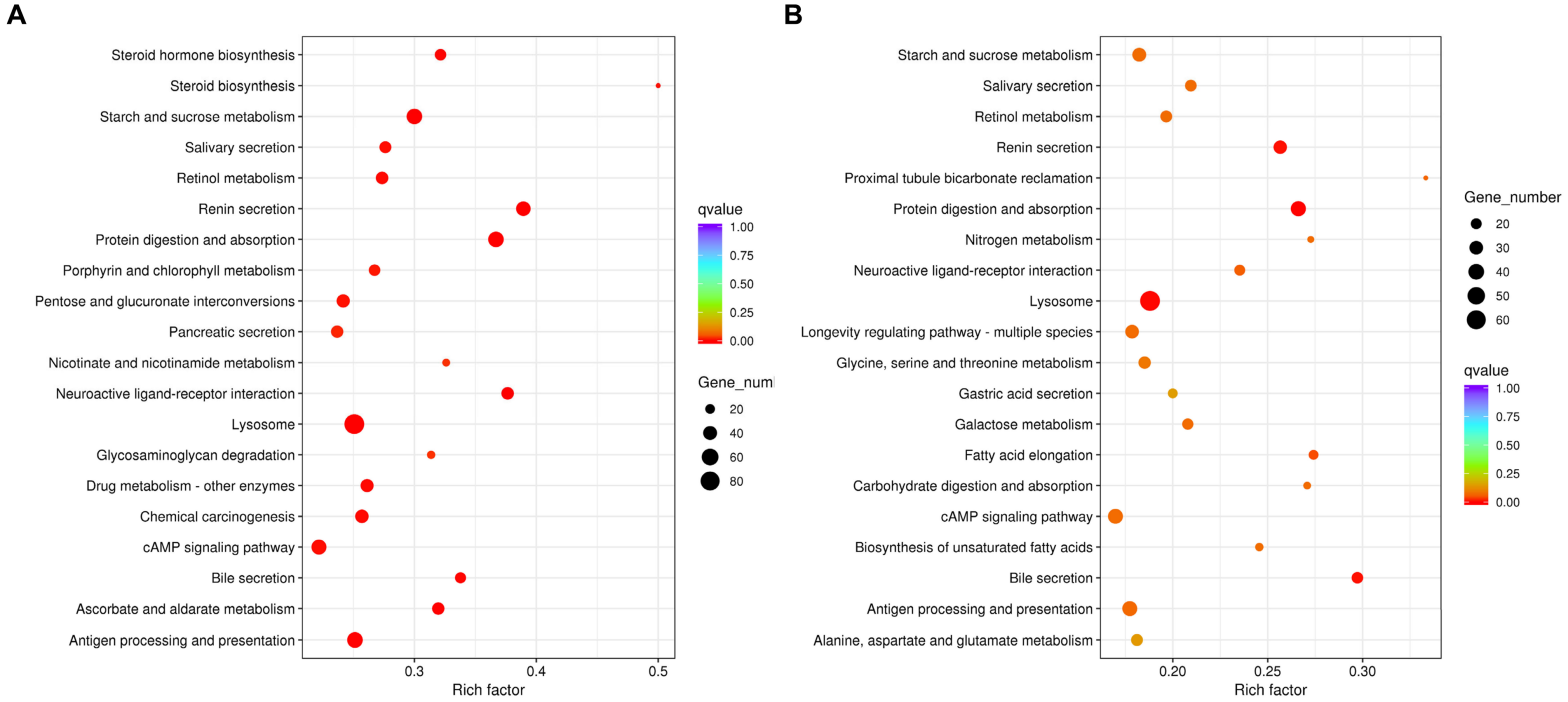
KEGG pathway analysis of AA vs. VA **(A)** and AM vs. VM **(B)**. Rich factor is the ratio of differentially expressed protein numbers annotated in this pathway. Greater rich factor (*p* < 1) means stronger intensiveness. *p*-value ranges from 0 to 1, and a lower p-value means greater intensiveness. Enriched pathway terms are displayed with *p* < 0.05. AA, aviruliferous Asia II-7; VA, viruliferous Asia II-7; AM, aviruliferous MED; and VM, viruliferous MED.

In addition, KEGG analysis revealed that DEGs of aviruliferous/viruliferous Asia II-7 were mostly involved in lysosome (92), purine metabolism (68), antigen processing and presentation (58), protein digestion and absorption (54), starch and sucrose metabolism (52), and the cAMP signaling pathway (50) (Figure 5). The DEGs of aviruliferous/viruliferous MED were mostly enriched in the lysosome (69), purine metabolism (48), protein digestion and absorption (37), antigen processing and presentation (37), and the cAMP signaling pathway (37) (Figure 5).

### 3.3 The highest enriched KEGG pathways of DEGs in VA vs. AA and VM vs. AM

**Figure 6:**
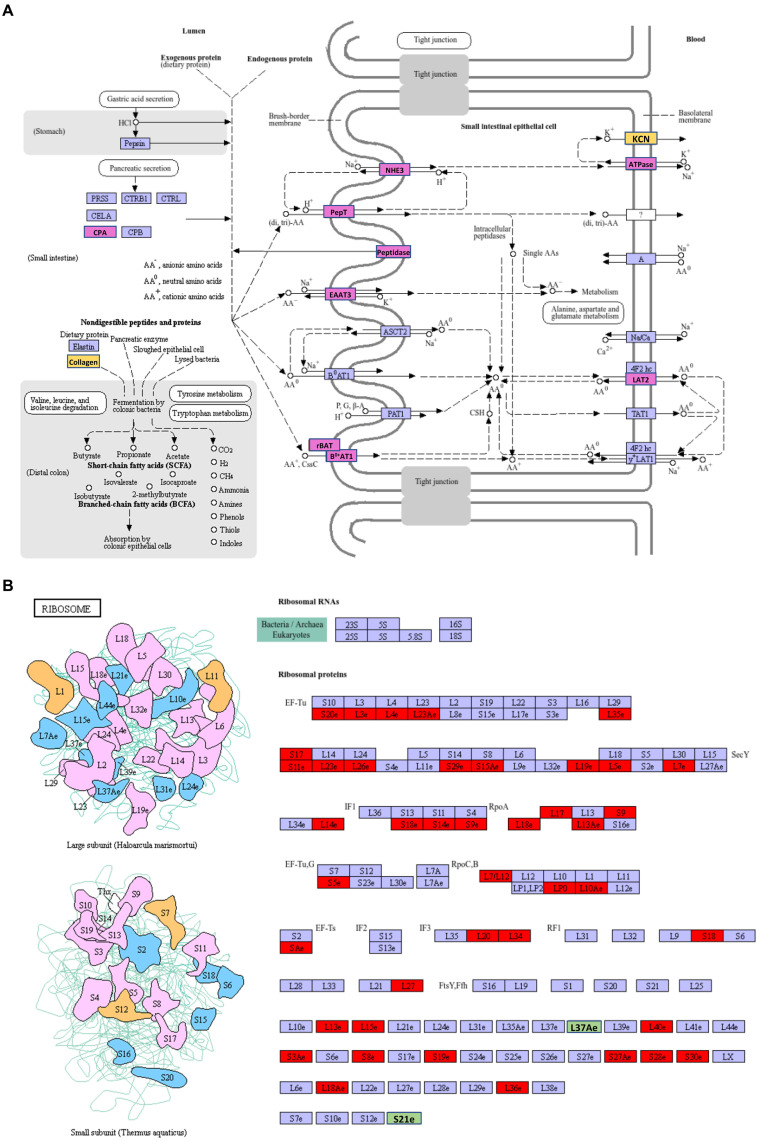
The highest enriched KEGG pathways of DEGs in VA vs. AA. **(A)** Upregulation DEGs; **(B)** downregulation DEGs. AA, aviruliferous Asia II-7; and VA, viruliferous Asia II-7. The purple or green box represents upregulation or downregulation in both viruliferous Asia II-7 and MED. The yellow or red box represents upregulation or downregulation only in viruliferous Asia II-7.

The most abundant upregulated DEGs of VA vs. AA in the KEGG pathway were protein digestion and absorption (Supplementary Table S3), which involves a complex series of degradative processes by enzymes in the digestive system to break down ingested protein into amino acids and small peptides. These components are then transported into enterocytes via various transporters and then used for amino acid metabolism or protein synthesis. When comparing aviruliferous whiteflies with viruliferous ones, the enzymes marked in purple were upregulated in both Asia II-7 and MED after CLCuMuV infection, suggesting a common response to the virus in these two species. Enzymes marked in yellow showed upregulation only in Asia II-7 after infection (Figure 6A). Overall, collagen and KCN were only downregulated in AA vs. VA, indicating a species-specific response to CLCuMuV. Collagen is the most abundant protein in the body. Its fiber-like structure is used to make connective tissue and is a major component of the bone, skin, muscles, tendons, and cartilage. The significant upregulation of collagen in viruliferous Asia II-7 indicated that the CLCuMuV might be impacting the connective tissue structure of the host and repair mechanisms of Asia II-7. “KCN” refers to a family of genes encoding potassium channels, which are crucial for maintaining the electrochemical gradient across cell membranes. Thus, upregulation of the KCN process in VA vs. AA might be a compensatory response to maintain cellular function, which was disturbed by CLCuMuV.

The most abundant downregulated DEGs of VA vs. AA in the KEGG pathway were ribosomes, which show the composition of ribosomes (Figure 6B). To our surprise, 44 ribosome compositions were downregulated in viruliferous Asia II, with only two of them (L37Ae and S21e) shared by both viruliferous Asia II and MED. The CLCuMuV impacted practically all ribosome biosynthesis activities, including initiation factors (IFs), elongation factors (EFs), release factors (RFs), and RNA polymerase subunits (Rpo), showing a deleterious impact on the ribosomes of Asia II.

### 3.4 Specific DEGs in Asia II-7and MED whitefly fed on CLCuMuV-infected cotton plants

**Figure 7:**
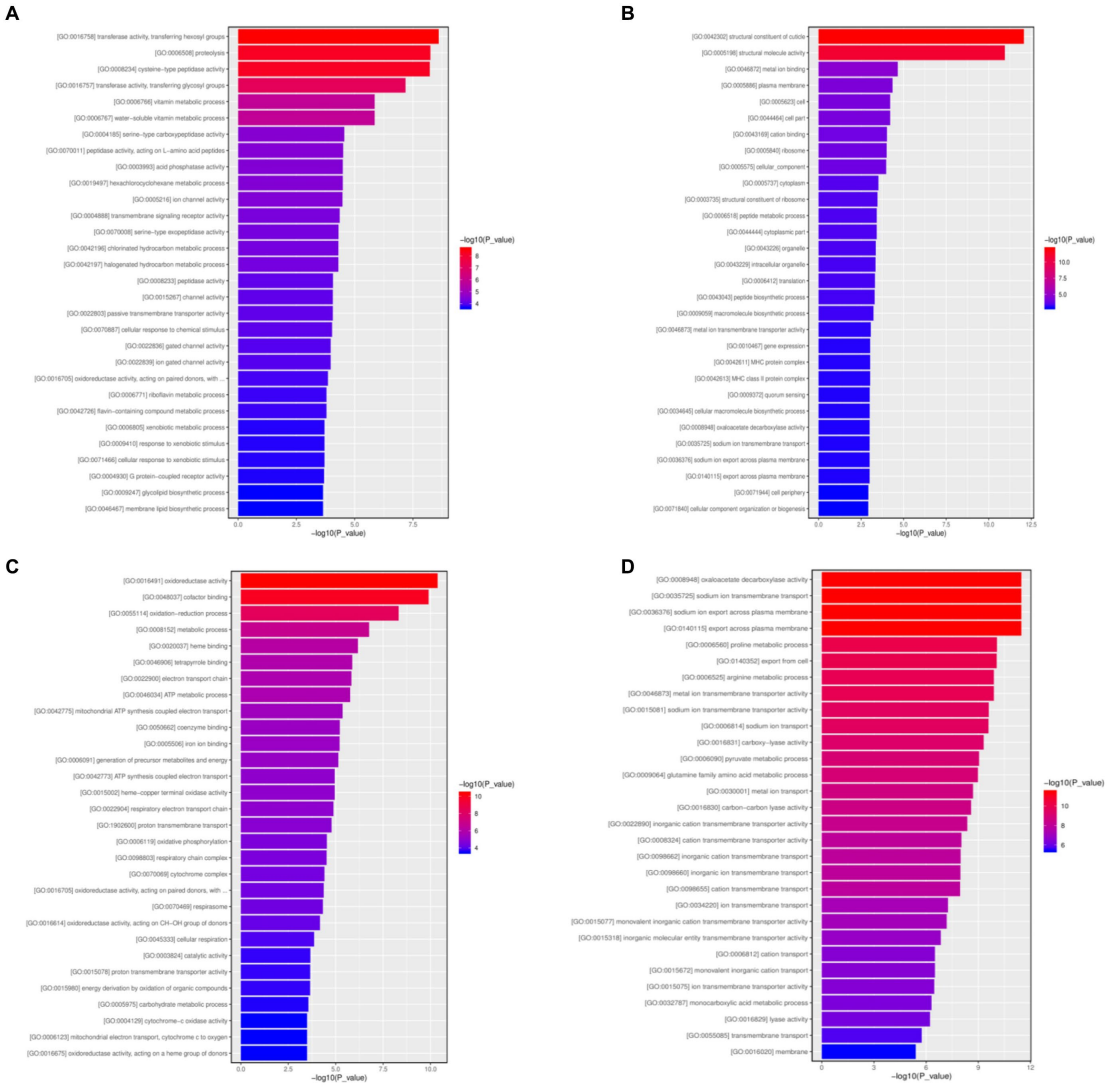
Specific DEGs in viruliferous Asia II-7 whiteflies (**A**, upregulated; **B**, downregulated) and in viruliferous Asia II-7 whiteflies (**C**, upregulated; **D**, downregulated) by gene ontology analysis. The bar chart shows the distribution of corresponding GO terms (*p* < 0.05). Different colors represent different GO categories.

A total of 8,119 specific DEGs in Asia II-7 MED whiteflies fed on CLCuMuV-infected cotton plants were identified, among which 4,342 genes were upregulated and 3,777 were downregulated. A total of 30 GO terms with the most significant enrichment were selected (Figure 7). In the biological process category, eight and nine items were assigned to upregulated and downregulated DEGs, respectively. Specifically, proteolysis, vitamin metabolic process, and hexachlorocyclohexane metabolic process were assigned to upregulated DEGs, while peptide metabolic, translation, and macromolecule biosynthetic processes were assigned to downregulated DEGs. As for the molecular function category, 22 items including transferring hexosyl groups, cysteine-type peptidase activity, and transferring glycosyl groups were associated with upregulated DEGs. On the contrary, seven items such as structural constituent of activity, structural molecule activity, and metal ion binding and transporter activity were assigned to downregulated DEGs. Finally, in the cellular component category, 12 items including plasma membrane, cell, and ribosome were linked with downregulated DEGs.

For MED whiteflies fed on CLCuMuV-infected cotton plants, a total of 2,892 specific DEGs were identified with 1,778 upregulated genes and 1,114 downregulated genes. In total, 30 GO terms with the most significant enrichment were selected (Figure 7). In the biological process category, 12 and 20 items were assigned to upregulated and downregulated DEGs, respectively. Oxidation–reduction process, metabolic process, and electron transport chain were assigned to upregulated DEGs, while sodium ion export across the plasma membrane, proline metabolic process, and export from cells were assigned to downregulated DEGs. In the molecular function category, upregulated DEGs were found in 13 items, such as oxidoreductase activity, cofactor binding, and hemebinding. On the contrary, downregulated DEGs were associated with 10 items, such as oxaloacetate decarboxylase activity, transporter activity, and carboxylase activity. As for the cellular component category, three items including respiratory chain complex, cytochrome complex, and respirasome were assigned to upregulated DEGs, whereas downregulated DEGs were linked with membrane.

### 3.5 Common response of Asia II-7 and MED whiteflies fed on CLCuMuV-infected cotton plants

**Figure 8:**
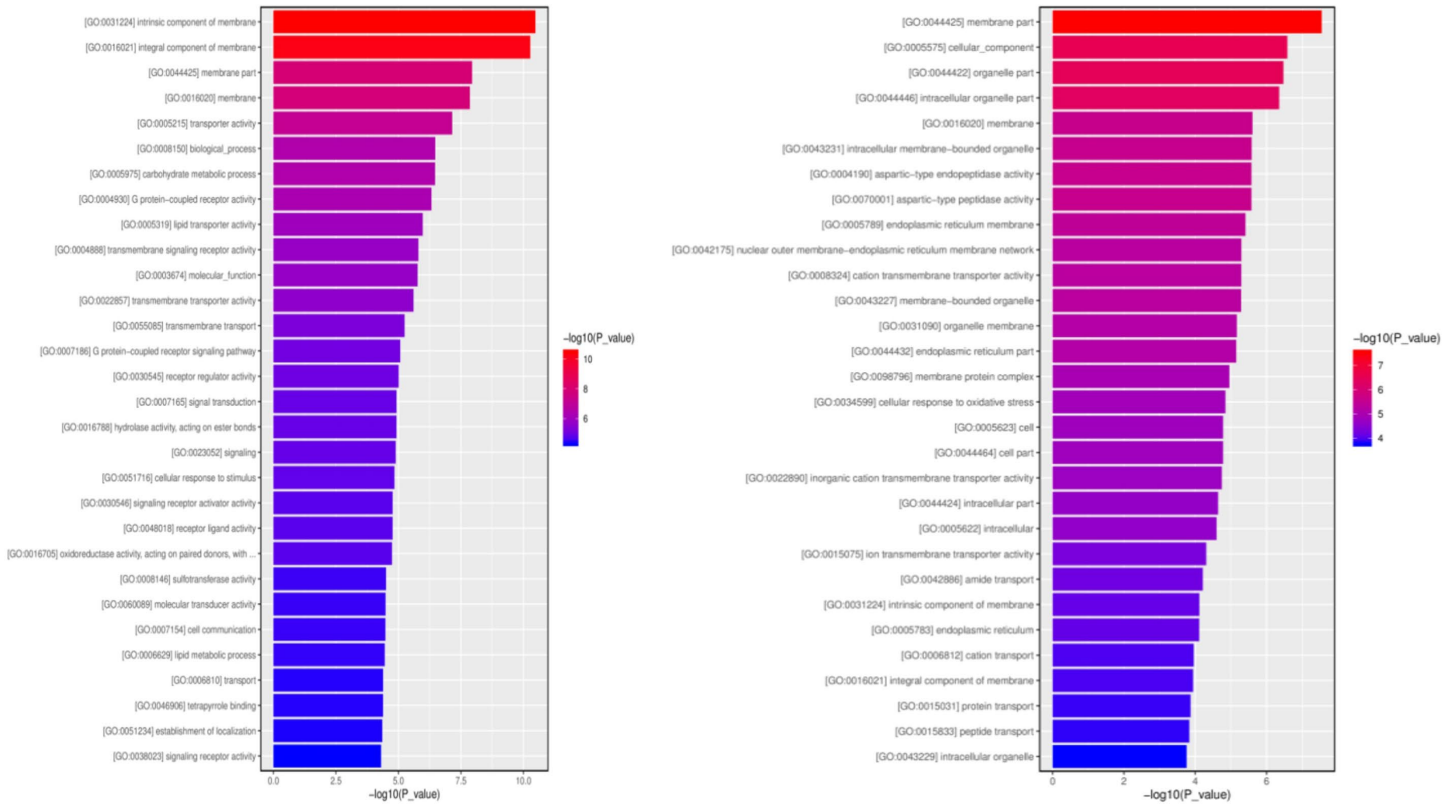
Common Response of Asia II-7 and MED whiteflies fed on CLCuMuV-infected cotton plants by gene ontology analysis. The bar chart shows the distribution of corresponding GO terms (*p* < 0.05). Different colors represent different GO categories. Left, upregulated; Right, downregulated.

A total of 6,094 common DEGs in Asia II-7 and MED whiteflies fed on CLCuMuV-infected plants were found. A total of 30 GO terms with the most significant enrichment were selected. As a result, most of the common DEGs in whiteflies either as vector species or non-vector species were associated with the membrane (Figure 8). In the biological process category, upregulated DEGs were found in 10 items including carbohydrate metabolic process, biological process, and transmembrane transport. Downregulated DEGs were annotated to four items, including cellular response to oxidative process and transport process, amide transport, and cation transport. In the molecular function category, 15 items including transporter activity, G protein-coupled receptor activity, and molecular function were assigned to upregulated DEGs. For comparison, downregulated DEGs were associated with five processes, including aspartic-type endopeptidase activity, aspartic-type peptidase activity, and cation transmembrane transporter activity. In the cellular component category, upregulated DEGs were assigned to 4 items, such as an intrinsic component of membrane, an integral component of membrane, and membrane, whereas downregulated DEGs were assigned to 12 items including the membrane part, cellular components, and the organelle part.

### 3.6 DEGs between VA vs. AA and VM vs. AM in cathepsin (CTS) biosynthesis

The CTS family of proteases plays a crucial role in the immune response, particularly through the process of antigen processing and presentation. We compared the DEGs between VA vs. AA and VM vs. AM in the CTS family (Table 1). Overall, 45 DEGs in the CTS family were identified in VA vs. AA, with 42 genes upregulated in viruliferous Asia II-7. The number of DEGs in the CTS family in VM vs. AM was much less, with 18 upregulated genes and 2 downregulated genes. Most DEGs were identified to belong to the CTSB family in both whitefly species.

### 3.7 The most abundant transcription factors between Asia II-7 and MED whiteflies induced by CLCuMuV infection

We further investigated and compared the TFs-related variations between Asia II-7 and MED whiteflies after CLCuMuV infection. Overall, 204 and 121 differentially expressed TFs, divided into 37 and 28 families, were observed in VA vs. AA and VM vs. AM, respectively (Supplementary Table S4). Most TFs in both whiteflies exhibited upregulated after CLCuMuV infection, with a proportion of 77.9% II-7 and 87.6% MED in Asia. The families with more than three DEGs are presented in Table 2. The zf-C2H2 TF family showed the largest number of DEGs, with 70 genes differentially expressed; of which, 37 were upregulated and 33 were downregulated in the AA vs. VA, and 34 DEGs were identified in the AM vs. VM. The TF families including homeobox, ZBTB, Forkhead, bZIP, and bHLH exhibited notable response patterns to virus infection, with more than 10 DEGs in AA vs. VA.

**Table 2:** The most abundantly detected differentially expressed TFs among whiteflies in response to CLCuMuV infection.

TF family	No. of DEGs			
	VAVA vs. AA up	VA vs. AA down	VM vs. AM up	VM vs. AM down
bHLH	9	1	8	0
zf-C2H2	37	33	19	15
ZBTB	17	1	7	0
THAP	5	1	1	0
TF_bZIP	9	1	9	0
T-box	3	1	1	1
PAX	4	0	1	0
Homeobox	23	0	18	1
HMG	4	3	5	0
Fork head	10	0	10	0
Total TF	159	45	106	15

TF, transcription factor; AA, aviruliferous Asia II-7; VA, viruliferous Asia II-7; AM, aviruliferous MED; VM, viruliferous MED.

### 3.8 RT-qPCR validation of DEGS

**Figure 9:**
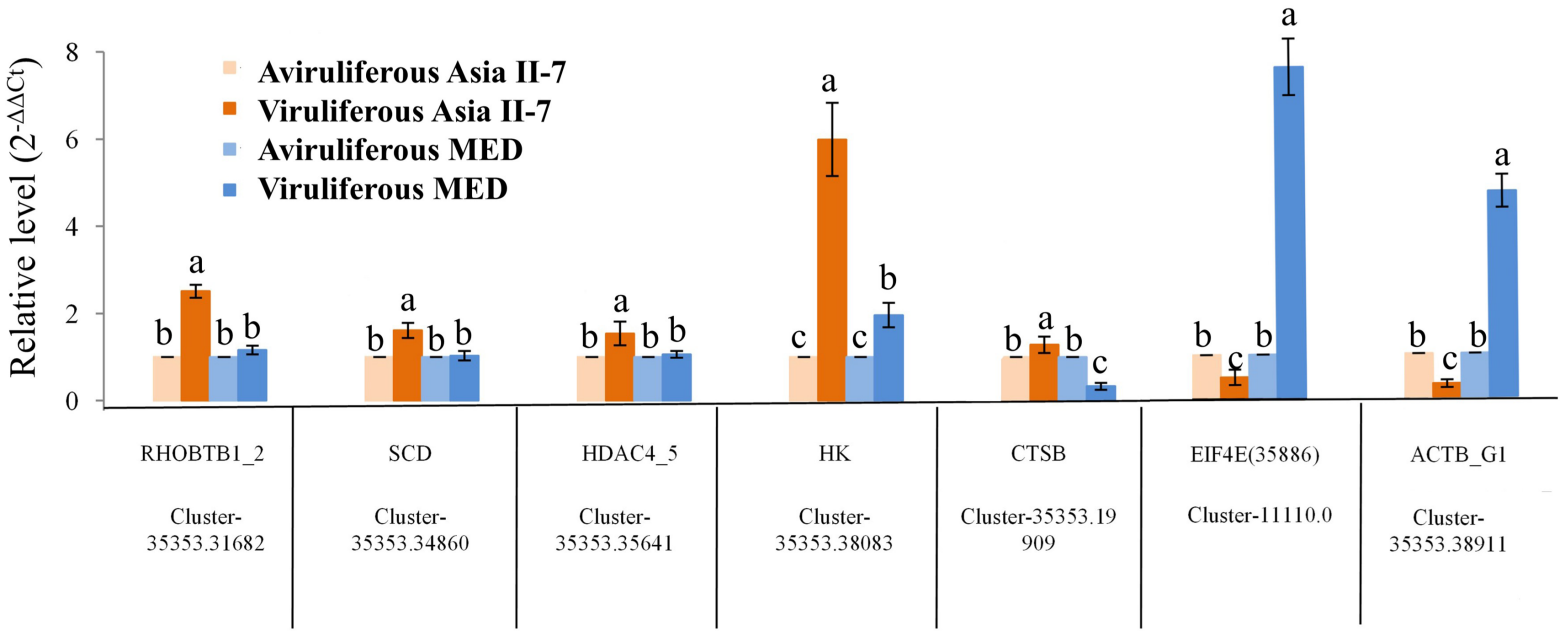
Expression patterns generated by the results of RT-qPCR. Five upregulated and two downregulated DEGs identified by RNA-Seq were validated by comparative CT (2^-ΔΔCT^) RT-qPCR with β-actin as the reference gene. All data were subjected to one-way analysis of variance (ANOVA) to determine the significant differences between aviruliferous and viruliferous whiteflies. Different small letters are significantly different at a probability level of 0.05.

The differential expression of seven selected DEGs was verified by RT-qPCR. As a result, five genes were upregulated in Asia II-7 whiteflies fed on CLCuMuV-infected cotton plants, while two genes were downregulated in Asia II-7 whiteflies. All these candidate genes were related to the immune and single-organism processes in aviruliferous/viruliferous Asia II-7 and aviruliferous/viruliferous MED. Specifically, Cluster-35353.31682 (RHOBTB1), Cluster-35353.34860 (SCD), Cluster-35353.19909 (CTSB), and Cluster-35353.38911(ACTB_G1) were important for the transport of *B. tabaci*, the activity of metabolism, and immune pathways. Other genes such as Cluster-35353.38083 (HK), Cluster-35353.35641(HDAC4_5), and Cluster-11110.0 (EIF4E) were associated with translation activity. Moreover, all seven genes showed the same expression pattern (upregulated or downregulated) for both RT-qPCR and RNA-Seq analysis (Figure 9; Supplementary Table S5).

## 4 Discussion

To investigate the interaction between begomoviruses and vector and non-vector cryptic species of whiteflies, gene expression profiles of viruliferous Asia II-7 (can transmit CLCuMuV) and MED (cannot transmit CLCuMuV) whiteflies were demonstrated in this study. Our results suggested the number of DEGs in Asia II-7 exposed to CLCuMuV (14,213) was higher than that exposed to MED (8,916) (Figure 4). The increased number of DEGs might indicate that Asia II-7 whiteflies activate a broader array of genes in response to the virus, to manage the infection or mediate transmission processes. Similarly, a study by Li et al. (17) suggested that the number of DEGs in *Sitobion avenae* exposed to *barley yellow dwarf virus* (BYDV) (a virus vectored by *S. avenae*) was higher than that exposed to *wheat dwarf virus* (WDV) (a virus not vectored by *S. avenae*). These results indicated that plant viruses might indirectly affect vectors compared with the effect on non-vectors. However, it still remains uncertain whether the increased number of DEGs was due to a stronger impact of CLCuMuV on Asia II-7 whiteflies.

Researchers have demonstrated that begomovirus infection induced transcriptional changes in the genes in whiteflies (17, 20, 21). Previous reports found that genes clustered in lipid, carbohydrate, and amino acid metabolism were downregulated in TYLCV-infected whiteflies (17, 18). It was also suggested that plant viruses could induce the fecundity and longevity of whiteflies, which might alter the expression patterns of genes involved in metabolic processes (19, 24, 25). In this study, GO enrichment analysis showed that the DEGs in viruliferous Asia II-7 and MED whiteflies were all highly enriched in metabolic process, transport, translation, and cytoskeleton (Figures 7, 8). This suggests that begomovirus infection influences similar metabolic pathways in different whitefly populations, supporting previous findings while also highlighting specific pathways that impacted our study.

Immune-related genes were important for resistance to begomovirus infection in whiteflies (26). Immune systems also impact virus replication and transmission by vectors. The previous study revealed that several immune pathways are vital for insects against viral infections, including Janus kinase, signal transducer and activator of transcription (JAK/STAT), Toll (27), immune deficiency, and c-Jun N-terminal kinase (28), and RNAi (29). In this study, when compared with MED whiteflies fed on viruliferous cotton plants, more immune-related genes were found upregulated in CLCuMuV-Asia II-7, such as cathepsin (CTS) family (Figure 6). Specifically, our data revealed that 45 DEGs in the CTS family were significantly expressed in VA vs. AA, with 42 of these genes upregulated. This contrasts with only 20 DEGs identified in the CTS family in VM vs. AM, highlighting a more robust immune response in Asia II-7 whiteflies. The increased expression of CTSB in Asia II-7 compared with MED was further validated by qPCR analysis (Figure 9), emphasizing the significant immune activation in this strain. The upregulation of CTS genes in our study indicates a potential mechanism through which CLCuMuV influences the immune system of whitefly. Li et al. (30) identified many DEGs associated with immune response, with five genes (including CTSB) upregulated in *S. avenae*-exposed BYDV. This alignment with our findings highlights the relevance of CTS genes in the immune response to viral infections. In viral infections, CTS can facilitate virus entry, replication, and spread by activating viral glycoproteins, processing antigens, and triggering cell death (31). There have been studies indicating an increase in the CTS families in whiteflies, which is linked to the whitefly’s immunological responses during the process of acquiring viruses or other interactions between whitefly and viruses (32). The dramatic upregulation of CTS genes in viruliferous Asia II-7 suggests that CLCuMuV has a significant detrimental impact on this whitefly population, potentially through heightened immune activation leading to increased metabolic costs or other deleterious effects. Viral transportation plays an important role in virus transmission, and whitefly cellular proteins affect the interaction between whiteflies and viruses (17). Whiteflies are non-vectors of some begomoviruses because of the presence of a selective transmission barrier at the luminal membrane surface of epithelial cells of the midgut (16, 33). Cyclophilin B and collagen (34), sugar transporter ERD6-like 6, ATP-binding cassette (ABC) transporters, and other proteins have been reported to influence viral transport (18, 20, 35). In this study, compared with AM vs. VM, specific upregulated DEGs in AA vs. VA were assigned to catalytic activity, binding, and transporter activity. Specifically, the expression of genes related to potassium channel protein was significantly upregulated in CLCuMuV- Asia II-7 (Figure 6). The subfamilies in KCN, such as KCNQ, KCNH, and KCNN, are important for the nervous system, cardiac tissue, and T-cell activation. It has been reported that the replication of the Israeli acute paralysis virus (IAPV) in bees can be regulated by potassium channel expression (36). Our data revealed that the expression of several potassium channel proteins was significantly elevated in CLCuMuV-infected Asia II-7 compared with their non-viruliferous counterparts. This upregulation suggests an adaptive response, where increased potassium channel activity could help mitigate cellular stress caused by viral infection. These proteins might influence the ionic balance and cell signaling pathways, which are critical during viral infection and replication. By contrast, the expression of ribosome components decreased significantly in VA vs. AA (Figure 6), which could be due to a strategy by the virus to suppress the host’s protein synthesis machinery, thereby reducing the ability of the host to mount an effective immune response (37). The significant downregulation of ribosomal proteins in viruliferous Asia II-7 whiteflies suggests that CLCuMuV might be manipulating the host’s translational machinery to favor its replication and persistence. By reducing the capacity of the host to produce proteins, the virus might be evading immune detection and response. Interestingly, the KCN and ribosome were not significantly affected in CLCuMuV-MED. These findings may help explain why MED has a lower viral transmission rate than Asia II-7.

Transcription factors are crucial regulatory proteins that control the expression of genes, including those involved in immune responses, cell cycle regulation, and stress responses. Moreover, our study focused on the TF regulated by CLCuMuV infection. Most differentially expressed TFs were upregulated in viruliferous whiteflies, with C2H2 ZF being the most abundant TFs (Table 2). The results were consistent with the previous report that the most abundant TFs among viruliferous whiteflies belonged to the zf-C2H2 family (38). It has been reported that ZAD containing C2H2 ZF proteins (ZAD-ZNF) constitute the most abundant class of insect transcription factors (39). Silencing of the ZF-like gene significantly increases virus loads retained within the whiteflies using three diverse begomoviruses (40). In our study, the upregulation of C2H2 ZF transcription factors in CLCuMuV-infected whiteflies suggests a critical role in the response of whiteflies to viral infection. Specifically, we identified that the expressions of multiple C2H2 ZF genes were markedly higher in viruliferous Asia II-7 whiteflies compared to their non-viruliferous counterparts. These transcription factors are likely responsible for controlling a group of genes that improve the defensive mechanisms of whitefly against the virus. This might possibly restrict the reproduction and dissemination of the virus inside the host. The substantial increase in the expression of these transcription factors may suggest a strong transcriptional response aimed at stimulating immunological pathways and other defensive mechanisms, enhancing resistance to the virus. Homeobox TFs were also abundant in whiteflies (Table 2). These genes are recognized for encoding proteins that include a homeodomain, which has significant functions in several developmental processes such as segmentation, differentiation, and control of the cell cycle. In fruit flies, the homeodomain protein POU regulates the continuous expression of antimicrobial peptide (AMP) genes, together with other regulators, to enhance the first defense against infection (41, 42). In this investigation, we have discovered 23 homeobox transcription factors that are expressed differently between VA and AA (Table 2). This finding suggests that homeobox genes play a role in the response to viruses. The differential expression of these homeobox genes suggests that they may be involved in regulating genes critical for antiviral responses, possibly through pathways that control immune function and stress responses. In the present study, we also identified 10 and 9 differently expressed bZIPs in VA vs. AA and VM vs. AM, respectively. BZIPs have significant functions in several biological processes in plants, such as development phases and the response to both abiotic and biotic stressors and signals (43). Nevertheless, the precise function of the bZIPs in insects remains unclear. Therefore, it is essential to conduct a functional analysis of whitefly transcription factors and the genes they control in future studies in order to get a comprehensive knowledge of the cellular processes involved in viral transmission.

## 5 Conclusion

In summary, we uncovered transcriptional responses of Asia II-7 and MED *B. tabaci* after feeding CLCuMuV-infected plants for 48 h. Our results indicated that CLCuMuV might impact vector cryptic species more than non-vector cryptic species because CLCuMuV-Asia II-7 had more DEGs (14,213) than CLCuMuV-MED (8,916). Genes involved in metabolism and immune systems were upregulated in viruliferous whiteflies. The most abundant upregulated DEGs of VA vs. AA in the KEGG pathway were protein digestion and absorption, while the most enriched downregulated pathway was ribosome. Furthermore, we identified specific genes in whiteflies related to cathepsin biosynthesis and transcription factors after virus infection. Overall, this study lays the foundation for further study on whiteflies and begomovirus interactions.

## Data Availability

The data presented in the study are deposited in the NCBI repository, the SRA accession number is PRJNA1144620.
