# Supplementary material for: Comparative transcriptome analysis of whiteflies raised on cotton leaf curl Multan virus-infected cotton plants
Source: Front Vet Sci. 2024 Aug 28;11:1417590. doi: 10.3389/fvets.2024.1417590 (PMC11389618; doi:10.3389/fvets.2024.1417590)
Supplement: Supplementary file 1 [file Table_1.DOCX]

Table S1 The primers information in this study

| **Gene** | **Forward sequence (5'-3')** | **Reverse sequence (5'-3')** | **PCR Products(bp)** |
| --- | --- | --- | --- |
| CLCuMuV | CAGGAAGCAGGAAAATACGAGA | TGGCAGTCCAACACAAAATACG | 837 |
| CLCuMuB | AAGTCGAATGGAACGTGAATGT | GGAGACCAAAAGAGGAGAGAGA | 831 |
| Cluster-35353.31682 | TGTAAAGGCTGGGTGGGATA | TGGAAGCAGTTGAAAAGGAGA | 111 |
| Cluster-35353.34860 | GGTAGTTGTGCCAGCCTTCG | CATCTTCGTCCGCTATGTCC | 155 |
| Cluster-35353.35641 | GTGGAACCAATGGAGGAAGAT | GCCGACAGTTAATGGGCTACT | 116 |
| Cluster-35353.38083 | TGCCACAGTTCAATCTGAGTTC | TATGTTCCTGAGTTGCCCAAT | 166 |
| Cluster-35353.19909 | AATTTCTACCCGCCGTCC | TTTCCCTATTTTCGCACTTTG | 134 |
| Cluster-11110.0 | TTGGTATGTGGAGCGGATGG | TATGCGGTGCTGTTGTCAGT | 197 |
| Cluster-35353.38911 | TCAACCATATTCGTCATCCAGT | TTTTGTTTGGCTTGCTGTGA | 105 |
| β-Actin | CATGGTCGGTATGGGTCAAA | GCTCTTCTGGGGCAACTCG | 173 |
