# Supplementary material for: Comparative transcriptome analysis of whiteflies raised on cotton leaf curl Multan virus-infected cotton plants
Source: Front Vet Sci. 2024 Aug 28;11:1417590. doi: 10.3389/fvets.2024.1417590 (PMC11389618; doi:10.3389/fvets.2024.1417590)
Supplement: Supplementary file 2 [file Table_2.DOCX]

Table S2. Summary of statistics for *Bemisia tabaci* transcriptome

| Statistics |  |
| --- | --- |
| Total number of transcripts | 275,505 |
| Total number of genes | 117,542 |
| Gene length > 1 kb | 28,289 |
| Mean length of genes | 952 |
| N50 gene length | 1,392 |
