# Supplementary material for: Comparative transcriptome analysis of whiteflies raised on cotton leaf curl Multan virus-infected cotton plants
Source: Front Vet Sci. 2024 Aug 28;11:1417590. doi: 10.3389/fvets.2024.1417590 (PMC11389618; doi:10.3389/fvets.2024.1417590)
Supplement: Supplementary file 6 [file Table_6.DOCX]

Supplementary Material

Supplementary Table 1 The primers information in this study

Supplementary Table 2 Summary of statistics for *Bemisia tabaci* transcriptome

Supplementary Table 3 The highest enriched KEGG pathways of differentially expressed genes in VA vs. AA.

Supplementary Table 4 The differentially expressed transcription factors between VA vs. AA and AM vs VM.

Supplementary Table 5 Transcriptomic data of selected genes.

**Supplementary Figure 1.** PCR analysis of CLCuMuV

Note：M：DL2000bp marker；1、3、5：non-viruliferous Asia II-7 whitefly；2、4、6：CLCuMuV-infected Asia II-7；7、9、11：non-viruliferous MED whitefly；8、10、12：CLCuMuV-infected MED；13、14：Mock
